# Supplementary material for: Saccharomyces boulardii (CNCM I-745) improves intestinal damage in sepsis by remodeling intestinal flora structure
Source: Front Cell Infect Microbiol. 2025 Jul 31;15:1602792. doi: 10.3389/fcimb.2025.1602792 (PMC12350329; doi:10.3389/fcimb.2025.1602792)
Supplement: Supplementary file 1 [file DataSheet1.zip › summary/summary/8_Advanced_analyse/2_PICRUSt2_result/COG/Group.DW0_vs_CW0_vs_BW0_vs_AW0/PICRUSt2_COG_Group.DW0_vs_CW0_vs_BW0_vs_AW0_diff.pdf]

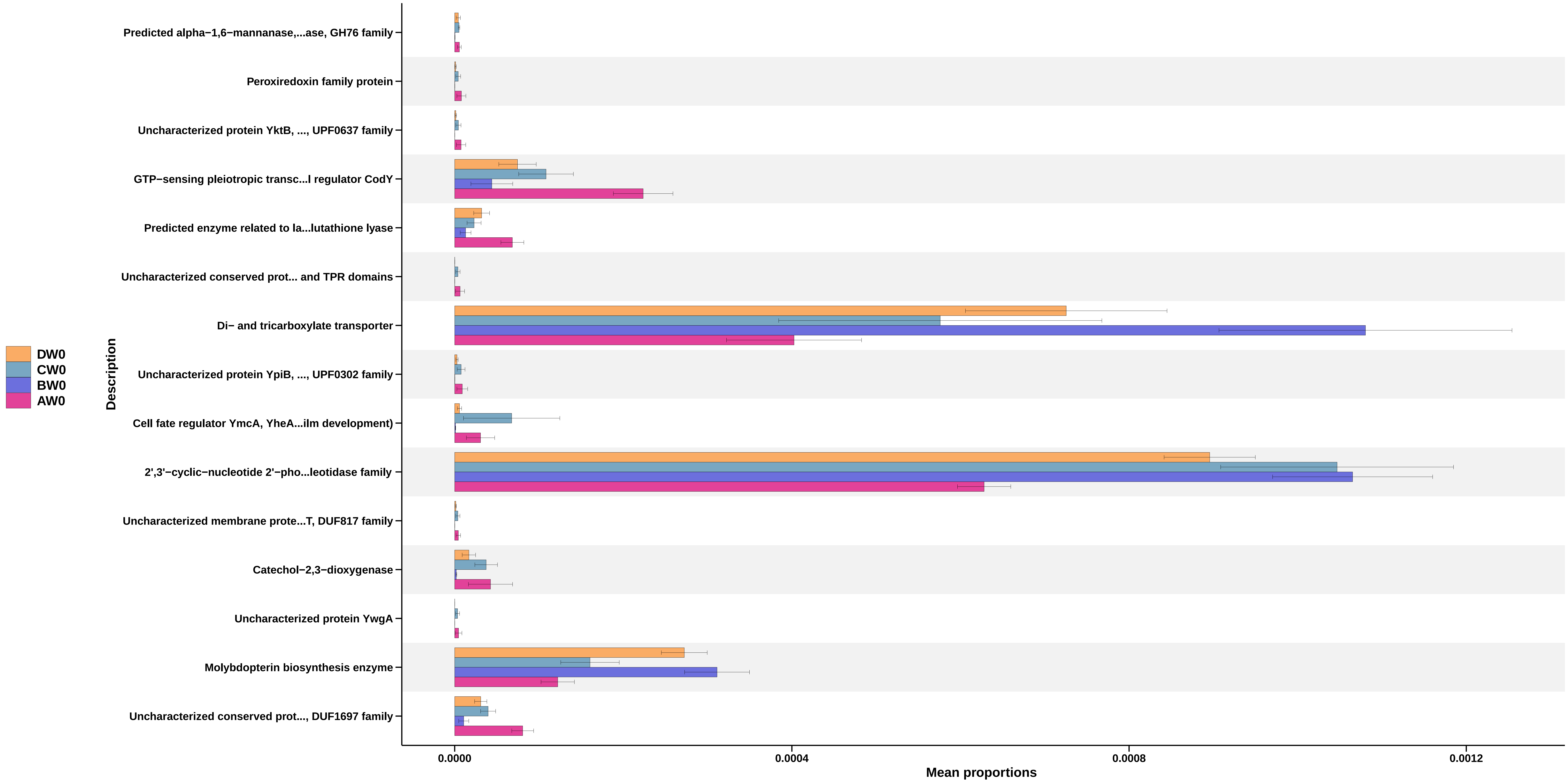

DW0
CW0
BW0
AW0
Description
Predicted alpha−1,6−mannanase,...ase, GH76 family
Peroxiredoxin family protein
Uncharacterized protein YktB, ..., UPF0637 family
GTP−sensing pleiotropic transc...l regulator CodY
Predicted enzyme related to la...lutathione lyase
Uncharacterized conserved prot... and TPR domains
Di− and tricarboxylate transporter
Uncharacterized protein YpiB, ..., UPF0302 family
Cell fate regulator YmcA, YheA...ilm development)
2',3'−cyclic−nucleotide 2'−pho...leotidase family
Uncharacterized membrane prote...T, DUF817 family
Catechol−2,3−dioxygenase
Uncharacterized protein YwgA
Molybdopterin biosynthesis enzyme
Uncharacterized conserved prot..., DUF1697 family
0.0000
0.0004
0.0008
0.0012
Mean proportions
